# A Mixed Methods Study on the Barriers and Facilitators of Physical Activity Associated with Residential Relocation

**DOI:** 10.1155/2018/1094812

**Published:** 2018-11-01

**Authors:** Grazia Salvo, Bonnie M. Lashewicz, Patricia K. Doyle-Baker, Gavin R. McCormack

**Affiliations:** ^1^Department of Community Health Sciences, Cumming School of Medicine, University of Calgary, Calgary, Canada T2N 4Z6; ^2^Faculty of Kinesiology, University of Calgary, Calgary, Canada T2N 1N4; ^3^Faculty of Environmental Design, University of Calgary, Calgary, Canada T2N 1N4

## Abstract

Despite evidence suggesting that neighbourhood characteristics are associated with physical activity, very few mixed methods studies investigate how relocating neighbourhood, and subsequent changes in the built environment, influences physical activity. This sequential mixed methods study estimates associations between changes in overall physical activity and transportation walking and cycling and changes in objectively assessed neighbourhood walkability (quantitative phase) and describes perceived barriers and facilitators to physical activity following residential relocation (qualitative phase). During the quantitative phase, self-reported changes in transportation walking, transportation cycling, and overall physical activity following residential relocation were measured using a 5-point scale: (1) a lot less now, (2) a little less now, (3) about the same, (4) a little more now, and (5) a lot more now. Walkability improvers reported a slight increase in transportation walking (mean = 3.29, standard deviation (SD) = 0.87), while walkability decliners reported little or no perceived change in their transportation walking after relocation (mean = 2.96, SD = 1.12). This difference approached statistical significance (*p*=0.053). Furthermore, walkability decliners reported a slight decrease in transportation cycling (mean = 2.69, SD = 0.96), while walkability improvers reported little or no perceived change in their transportation cycling after relocation (mean = 3.02, SD = 0.84). This difference was statistically significant (*p* < 0.05). Change in walkability resulting from relocation was not significantly associated with perceived change in overall physical activity. Our qualitative findings suggest that moving to a neighbourhood with safe paths connecting to nearby destinations can facilitate transportation walking and cycling. Some participants describe adjusting their leisure physical activity to compensate for changes in transportation walking and cycling. Strong contributors to neighbourhood leisure physical activity included the presence of aesthetic features and availability of recreational opportunities that allow for the creation of social connections with community and family.

## 1. Introduction

In recent decades, increasing research and political interest have focused on interventions that modify neighbourhood built environments to facilitate physical activity [1]. However, evidence informing these interventions is mainly derived from cross-sectional studies [2, 3]. Cross-sectional studies cannot provide temporal evidence and are vulnerable to biases that may result in spurious associations, making it difficult to infer causality between the built environment and physical activity [4, 5]. Quasi-experimental and -longitudinal studies are vital to illuminating causal relationships between the built environment and physical activity [5]. Despite these more rigorous study designs providing estimates of temporal relations between the built environment and physical activity [6, 7], they provide limited evidence about why, how, and under what conditions, these relationships exist. Qualitative and indeed a mix of qualitative and quantitative research approaches are needed to understand the pathways by which the built environment is associated with physical activity.

Quantitative research findings suggest that neighbourhood connectivity, residential density, land use mix, diversity of destinations, and indices of overall walkability including Walk Score® (a publicly available, objectively derived walkability indicator) are associated with physical activity in longitudinal residential relocation studies [8–10]. Hirsch et al. found that participants moving to a neighbourhood with a higher Walk Score® reported an increase in transportation walking but reported no change in their leisure walking. Furthermore, changes in neighbourhood safety, availability of outdoor spaces, aesthetics, access to public transportation, and physical activity opportunities have been associated with perceived physical activity change in quasi-longitudinal residential relocation studies [11–13]. Others have found perceptions of the neighbourhood built environment to be associated with changes in walking and cycling [14, 15]. Compared with objectively determined access to destinations, perceived access to destinations may have greater impact on adult's decisions to walk for transportation [7, 16].

Qualitative research has explored how built characteristics such as safety, functionality, destinations, and aesthetics influence physical activity preferences [17–19]. Nevertheless, such studies call attention to barriers and facilitators to physical activity specific to sociodemographic groups [19]. For example, functional features such as railings, benches, washrooms, and shade along pathways encourage walking among older adults [20–25] while fear of crime may be a barrier to outdoor physical activity especially among socioeconomically disadvantaged populations [26–30]. Specific aesthetic features, including greenery, parks, and gardens, may motivate walking in adults as these features confer feelings of peace and restoration [19, 22, 24, 31–34]. Although one qualitative study found that changes in commuting habits following relocation was motivated by convenience, speed, cost, and reliability of the transportation mode [35], few qualitative studies explore how changes in the built environment following relocation impact physical activity. A qualitative study of adults who have relocated neighbourhood will generate participant descriptions and comparisons of how and why their experiences being physically active change in different lived environments.

This sequential mixed method study has two objectives: (1) estimating the associations between perceived changes in walking and cycling for transportation and overall physical activity and changes in objectively assessed neighbourhood walkability (Walk Score®) and (2) describing perceived built environment barriers and facilitators to physical activity following neighbourhood relocation. Informed by previous evidence [8–10], we hypothesized that improvements in objectively assessed walkability would be associated with a perceived mean improvement in transportation walking and transportation cycling when compared with decrease in objectively assessed walkability. Through these objectives, we provide novel evidence about individual, social, and environmental factors that potentially influence changes in physical activity following a residential relocation.

## 2. Materials and Methods

### 2.1. Study Design

Our mixed methods sequential study design included two phases, and we placed greater emphasis on qualitative findings that help interpret our quantitative results [36]. Phase I is a quantitative descriptive analysis of existing survey data on changes in physical activity behaviours and changes in the built environment following residential relocation. Phase II is a narrative study, specifically, a qualitative analysis of narratives, through which we explore participant reasons for the behaviour change following residential relocation. At its foundation, narrative research is about collecting and describing participants' stories, or “consequential linking” of experiences [37], and then interpreting the meanings of those experiences. Stories provide access to the richness of experience because people use stories to reflect who they are, what they have experienced, and what and how they wish to share their “internalized world” with others [38]. Thus, through stories, researchers may obtain in-depth insights into participants' experiences. In our mixed methods study, analysis of narratives is used to add meaning and understandings of lived experiences to help explain the changes in physical activity-related behaviour evident in the quantitative results.

### 2.2. Sample Design and Participant Recruitment

In 2014, a large stratified random sample of Calgary households (*n* = 10,500) were sent survey packages, of which *n* = 1023 adults completed online and postal surveys. A full description of the sampling and data collection are detailed elsewhere [39, 40]. For this study, we drew upon *n* = 113 participants who reported relocating neighbourhood in the past 12 months. Of these, 16 moved to a neighbourhood of same walkability (based on Walk Score®), 49 moved to a less walkable neighbourhood, and 48 moved to a more walkable neighbourhood. For our quantitative analysis (Phase I), we included data from all 97 survey respondents who experienced a change in neighbourhood walkability after the relocation. For the qualitative analysis (Phase II), we reached out to 42 survey respondents who had relocated neighbourhood and agreed to be contacted for future research, of which 14 agreed to participate in a semistructured interview. This sampling strategy allowed us to capture, compare, and contrast adult's perceived barriers and facilitators to physical activity in relation to their experience residing in two physically different neighbourhood environments. The University of Calgary's Conjoint Health Research Ethics Board approved this study. Verbal informed consent from participants was obtained prior to commencing interviews. Anonymity of participants was protected by assigning pseudonyms, eliminating identifying information, and paraphrasing content.

### 2.3. Data Collection

Phase I: data collection via online and postal surveys was completed in 2014 [39, 40]. Sociodemographic variables relevant to the current study captured from the survey included sex, age, education level, dog ownership, and presence of injury that impacted walking. Physical activity variables included respondents' perceived change in their transportation walking, transportation cycling, and overall physical activity since their relocation. Response options for these three physical activity items were captured on a 5-point scale: (1) a lot less now, (2) a little less now, (3) about the same, (4) a little more now, and (5) a lot more now. Perceived quasi-longitudinal changes in physical activity have been captured with similar response options elsewhere [11, 12] and have acceptable test-retest reliability [13]. Participants reported their previous and current neighbourhood name and/or postal code. Walk Score®, available for 6-digit Canadian postal codes, was aggregated to the neighbourhood administrative level and used to estimate the walkability of the participants' previous and current neighbourhoods. Aggregation of Walk Score® was undertaken because not all movers could accurately recall the postal code of their previous households, but all could recall the name of their previous neighbourhood. Due to the date the quantitative data were collected, we used the previous version of Walk Score® (estimated based on distance to 12 destinations posited to be important for walking). Notably, the previous version of Walk Score® did not capture built characteristics such as personal safety, aesthetics and attractiveness, and streetscape pedestrian infrastructure, all of which may be associated with leisure walking and other physical activities [41–43].

Phase II: qualitative data were collected in 2016 through individual semistructured telephone interviews each lasting approximately one hour. Our interview guide included questions to elicit information about individual, social, and environmental barriers and facilitators to physical activity, and although given the focus of this study, our emphasis was on understanding the neighbourhood built environment determinants. We used an existing conceptual framework to develop interview questions related to the built environment. This framework organizes the potential environmental influences on walking and cycling into four constructs: aesthetics (i.e., cleanliness, greenery, and pleasant sights), functionality (i.e., direct routes, path maintenance, and street design), destinations (i.e., local facilities, services, and public transportation), and safety (i.e., traffic and crime safety) based on previous literature and expert consultation [44]. This conceptual framework has been used to guide extraction and reporting of the built environment and physical activity findings in both qualitative [45] and quantitative studies [45–47]. The interview guide was pilot tested internally to ensure that questions were understandable and likely to elicit rich descriptive responses. A trained graduate student conducted interviews, which were audio-recorded and transcribed verbatim.

### 2.4. Data Analysis

Phase I: Walk Score® change between participants' previous and current neighbourhoods, regardless of the magnitude, was used to dichotomize participants as being a “walkability improver” or “walkability decliner.” Due to the small sample, we were unable to undertake a sensitivity analysis to test the influence of other Walk Score® cut-points for identifying walkability improvers or decliners. We analyzed perceived change in each physical activity as numerical outcomes using the original 5-point scale (i.e., values equal to 3 indicated no perceived change, values below 3 indicated a perceived decrease, and values above 3 indicated a perceived increase in physical activity).

We estimated descriptive statistics and frequencies for the sample sociodemographic characteristics (i.e., gender, dog ownership, motor vehicle access, marital status, education level, presence of dependents, and presence of injury) stratified by the residential status (i.e., walkability improver vs walkability decliner). We used independent *t*-tests to assess the relations between perceived change in transportation walking, transportation cycling, and overall physical activity and changes in walkability (“improvers” vs “decliners”). Informed by previous quantitative evidence [10], we used one-tailed *p* values to test our directional hypothesis that increasing walkability leads to increased physical activity after relocation, and decreased walkability leads to decreased physical activity after relocation. We use a significance level of alpha = 0.05, and SPSS version 22 (SPSS Inc., Chicago, IL, USA) was used to analyze our quantitative data.

Phase II: our narrative informed data analysis [48–50] is consistent with Polkinghorne, as we relied on stories that have a linear timeline including beginning, plot, and denouement that allow us to understand present choices by linking them to prior events, specifically how residential relocation influenced physical activity behaviour change. We applied a three-dimensional space approach [51] to analyze data from three dimensions: interactions (social and individual), continuity (time before and after the move), and situation (physical environment). The interview transcription, handwritten interviewer notes, and coding were managed electronically using NVivo 10.2.2 software. Analysis was iterative and occurred alongside data collection in multiple stages [52]. First, long interview segments were separated into individual stories by identifying scenes in which a plot unfolds and by scanning for story characters and chronology. Each story was then scanned for themes. We then rescanned all stories and recordings for other dimensions that may have been missed upon first reading such as emotional, interpersonal, and cultural dimensions. Next, the stories were examined for commonalities and differences to allow us to identify themes, patterns, and differences between stories. Differences between stories were then examined in relation to the different environmental settings and sociocultural contexts in which the participants are living. Themes were discussed and refined among the coauthors. For the analysis of built characteristics, interviews were coded according to expected themes (functional characteristics, safety, destinations, and aesthetics). Characteristics that did not fit into the expected themes were coded separately.

Efforts to enhance the trustworthiness of our study began with “member checking” with participants during interviews and were followed with “peer debriefing” by developing and refining themes among coauthors [49]. To enhance transferability, the particularities of each story were described to illuminate context and relevance to other settings while dependability was enhanced by maintenance of an audit trail detailing the logic of data analysis decisions [48].

## 3. Results

### 3.1. Quantitative Findings

We found no significant differences in the sociodemographic characteristics of walkability improvers and walkability decliners (Table 1). On average, walkability improvers reported a slight increase in transportation walking, while walkability decliners on average reported little or no perceived change in their transportation walking after relocation. This difference approached statistical significance (walkability decliner 2.96 ± 1.12 vs 3.29 ± 0.87, *p*=0.053) (Table 2). We also found that, on average, walkability decliners reported a slight decrease in transportation cycling, while walkability improvers on average reported little or no perceived change in their transportation cycling after relocation. This difference was statistically significant (walkability decliner 2.69 ± 0.96 vs 3.02 ± 0.84, *p*=0.039). Change in walkability was not associated with perceived change in overall physical activity, although both walkability improvers and decliners reported increases in overall physical activity after the move.

### 3.2. Qualitative Findings

Our 14 interview participants were 12 women and two men. Nine participants increased walkability (“improvers”) while five decreased walkability (“decliners”) after relocating neighbourhood (Table 3). We generated three broad themes of influences on changes in physical activity after relocating: (1) the built environment and getting “around,” (2) neighbourhood opportunities that offer “a chance to connect,” and (3) adjusting or adapting physical activity behaviours in response to a new environment. The built environment and getting “around” theme were comprised of four subthemes: (a) functional features (i.e., street pattern preferences), (b) safety features (i.e., exposure to traffic and feelings of security), (c) aesthetic features that support versus not aesthetic features that act as barriers to physical activity, and (d) nearby destinations that encourage active transportation choices. The second theme neighbourhood opportunities that offer “a chance to connect” was divided into two subthemes: (a) characteristics that enable physical activity while connecting with family and community and (b) characteristics that allow for connections with nature and feelings of sustainability. The third theme of adjusting or adapting physical activity behaviours in response to a new environment describes how different built characteristics allowed for changes in transportation and leisure physical activity.

### 3.2.1. The Built Environment and “Getting Around”

*(1) Functional Features*.

- Street pattern preferences according to physical activity context

Some participants report that grid pattern streets allow ease of access from point A to B (i.e., improving way finding). Marie describes her new more walkable neighbourhood by noting: “I live in kind of old-fashioned streets where they go straight north and south, east and west so you don't have to go out of your way to get somewhere.” Although some participants appreciate having relocated to grid-like neighbourhoods for ease of getting around via active transportation, others prefer curvilinear street patterns as these streets allow exploration of different areas during leisure time walking, as Alexia explains: “The roads are not as uniform, so I find I can explore a little bit more when I walk through this neighbourhood rather than the other neighbourhood. (…) it's a bit more enticing I guess. […] there's more parks with cul-de-sacs, there's unusual walking roads, there's different options rather than the other house I was just living on a street and I [could go] to the left or right.” These participants suggest that grid pattern neighbourhoods may support transportation walking while curvilinear street patterns could support leisure walking and cycling.

*(2) Safety*.

- “Not very pedestrian friendly”: high traffic areas that encourage car use

In some neighbourhoods, the presence of large boulevards encouraged participants to use their cars instead of walk. They feel that car drivers are not looking out for pedestrians or cyclists at intersections. In areas of high traffic, having crossings separated from roads, such as pedestrian bridges made participants feel somewhat safer. Melina, who enjoys transportation walking in her current more walkable neighbourhood, describes having rarely walked to amenities in her old neighbourhood as she had to cross a large, busy boulevard: “[In previous neighbourhood], I was definitely less active just because I lived beside a main thoroughfare so it's definitely not safe for cycling and I just found it much easier to get around there by car than by cycling (…) it's not very pedestrian friendly to cross [the thoroughfare]. It's dangerous, it's loud, you have to run across the boulevard to make sure that you get across in time (…) it would be better if there was a pathway or a bridge going over.” Participants explain that pathways that are separate from motorized traffic and connected to destinations of interest allow them to feel safer and increase their walking and cycling for leisure. Denise (walkability improver) notes that in her previous neighbourhood “there weren't as many walking paths or quiet areas, [it] was a lot busier with a lot more traffic so I tended not to go out as much. Whereas in [my current neighbourhood], it's a lot quieter being closer to [a large provincial park], walking distance to stores and so I find I'm a lot more physical in this neighbourhood than I was.”

Cyclists find that separating bike paths from traffic is both safer and less exhausting as noted in Eva's description of traffic lights in her current more walkable neighbourhood: “to stop on a bike and then to keep going takes a lot of energy (…) It's more encouraging if you don't have to ride with the traffic. I used to ride on [the street] but it was really miserable […] Now, I've rerouted to the pathways along the river so it's more enjoyable, it definitely encourage [s] more physical activity.” Relocating to a more walkable neighbourhood, and gaining access to pathways separate from motorized traffic, encourages participants to use active modes of transportation on the grounds of safety afforded by pathways, bike lanes, and cycle tracks.

Participants who relocated to neighbourhoods with a limited number of motor vehicle entry points appreciate how this feature offers protection from high traffic volumes and supports physical activity in a peaceful environment. Denise [a walkability improver] says “[My new neighbourhood] is safer because there are only two entry points so that helps to cut down on traffic […]. Whereas in [my previous neighbourhood], there was definitely a lot more traffic […] I don't like being around traffic, […] so I […] didn't get out as much in that (previous) neighbourhood. Whereas here, I'm more inclined to go out because I know there won't be as much traffic.” Exposure to built characteristics that reduce interactions with traffic by diminishing neighbourhood traffic encourages increases in both leisure and transportation walking and cycling while supporting a more pleasant experience.

(ii) “Confidence in the community”

Feelings of security are important in participant descriptions of expanding their outdoor exercise hours after dark in neighbourhoods with which they are familiar. Bernadette describes feeling safer in her new, less walkable neighbourhood given the confidence she has in her neighbours: “I can easily walk during the day and the evening or even at night, I don't really have anything that I'm concerned about […] because of that confidence I have with the community, I feel it is a safe place.” Participants do not report changes in the actual level of criminal activity in their new neighbourhoods as making them change their physical activity, but rather speak in terms of confidence in their new neighbours as determining their physical activity habits.

*(3) Aesthetics*.

- “It just feels better”: physical activity near greenery and nice surroundings

The presence of nature or natural features is a motivator for neighbourhood PA. Participants who relocated from neighbourhoods that contained smaller parks to neighbourhoods with larger parks report that the larger parks increased their walking for leisure. Participants indicate being drawn to greenery and nature and prefer to be physically active amidst nature during their leisure time for the calmness, peace, and quiet nature provides. Chrissy (a walkability improver) reports “It just feels better to have nice lush trees and a nice surrounding. It's more encouraging to want to go out.” Large parks are described by Denise (a walkability improver) as offering nature's restorative properties: “It's a lot quieter, there's a lot more wildlife [in new neighbourhood] […] to me that makes it a lot more peaceful [than previous neighbourhood).” Mature trees lining streets, community and private gardens, yards, and parks are mentioned as spaces for connecting with nature. Participants also claim that, compared to newer neighbourhoods/areas with many rental properties, in older neighbourhoods, homeowners tend to have more pride in ownership and are likely to have invested in features such as varied architecture and gardens which can motivate participants to walk for leisure and enjoy surroundings. Alexia (walkability improver) describes her increased walking behaviour in an architecturally mixed neighbourhood: “unique houses in this area. There's small ones and big ones. Whereas the other [neighbourhood] was more smallish houses. [This neighbourhood is] more interesting […] I enjoy walking around and looking at the different things that people have done to their homes so I would say it does [motivate me to walk more].” For many participants, having relocated to neighbourhoods with large parks and attractive or interesting views or scenery increased leisure walking.

(ii) “Too many cars”: unaesthetic barriers to physical activity

Participants report the presence of cars as unattractive and discouraging to spending time outside. Specifically, cars parked on lawns and the presence of large parking areas make neighbourhoods less attractive and discourage walking. Eva explains that in her current more walkable neighbourhood “The city came by this year to prune all the trees. It looked really nice […] The streets are wider, they're not cluttered with parked cars [as in previous neighbourhood].” Proximity to cars is also unpleasant because of air and noise pollution. Bernadette (a walkability decliner) notes “At the same time I find [my new neighbourhood] a little bit [more] quiet, because I'm not on the ring road like [in my previous neighbourhood] I don't like too much noise and too many cars”. Pedestrians and cyclists both express discomfort engaging in physical activity near infrastructure designed for motorized vehicle use as they consider car exhaust and parking areas unpleasant.

*(4) Destination*.

- “Just because it is close”: nearby destinations' influence on active transportation

Participants appreciate leisure destinations such as parks, recreational facilities, cafés, and friends' houses, to be in walking distance from home. Some participants enjoy cycling to work, some use a combination of walking and public transportation, and others drive their private motor vehicles. Most prefer running errands, such as grocery shopping, by a car given the inconvenience or physical challenge of carrying bags of groceries. For example, Eva (a walkability improver) started cycling for small errands following relocation but still utilizes her car occasionally for groceries: “But for major groceries, I guess it's easier to bring back food [by a car].”

For participants who had previously used active transportation, cycling activity decreased after resettling in lower walkability neighbourhoods with fewer amenities and where car culture was prioritized. Laura (a walkability decliner) points out “[In previous neighbourhood] they have the paths that are accessible for riding your bikes […] but here you don't see that nearly as much, we're so used to driving our cars places and stuff so it's the whole culture that's already set up like that.” However, those who relocated to neighbourhoods with increased walkability were able to change their behaviours and use more active transportation as described by Melina (a walkability improver): “So definitely, I'm more active in [current neighbourhood] just because it is close to downtown, it's easy to cycle to the grocery store or downtown or to a friend's house, and some things are walking distance.” Notably, some participants report starting to use public transit and thus encompassing daily walking to get to work after moving to a more walkable neighbourhood.

Most participants who improved walkability appreciate destinations near their new homes that encourage them to walk or bike rather than using the car. However, personal characteristics and motivation may lead to increased physical activity in less walkable environments where longer distances need to be traveled. For instance, Emilia (a walkability decliner) notes that because she is retired and no longer drives, she has more time available to take longer walks to local destinations: “I walk to my dentist because I don't have a car, and besides I'm diabetic, I prefer to walk ‘cause I figure that's better than being in a car anyway. So not having a car, it's just forced me into longer walks (laughter) and more often sometimes.” Participants also enjoy actively commuting to neighbourhood recreation destinations including gyms, tennis courts, the library, and local cross-country ski areas. Having such destinations within walking or cycling distance from home could support physical activity, especially where the motivation and time to actively travel to further destinations are lacking.

### 3.2.2. Neighbourhood Opportunities That Offer “A Chance to Connect”

*(1) Connections with Community and Family*. Those who maintained regular physical activity habits often use physical activity as a conduit for connections with others and their environments. For example, two participants changed their physical activity habits while building connections with colleagues. Becky (a walkability improver) describes “colleagues definitely encourage me to be active (…) as I've been transitioning into biking to work. It helps!” Regardless of their neighbourhoods' walkability, participants value leisure walking within their neighbourhoods for potential to meet others. Rosa (a walkability decliner), finds herself walking more for leisure in her new neighbourhood because she feels more engaged in its social fabric than in her previous more walkable neighbourhood: “It's nice going for a walk and meeting someone from the neighbourhood that you know, saying hello, getting engaged with others.” However, neighbourhoods where social connections are lacking can offer less incentive to walk as highlighted by Marie (a walkability improver): “I guess you could walk to anywhere but, I did not care to do it there [in previous neighbourhood].” Certain neighbourhood spaces support social connections; for example, large parks can provide socializing spaces for adults. Bernadette (a walkability decliner) expresses missing her old neighbourhood because of the social space the large park created: “The [large park] was more of a social place for people [who] used to play games, they used to play soccer […] It was a big park so people would go there, and enjoy themselves.” Physical activity connects community members and family members. Parents describe changing their physical activity patterns depending on the age and activities of their child(ren). Laura (a walkability decliner) claims that after her daughter grew up and moved out of the house, her physical activity levels decreased: “In [my previous neighbourhood], I used to ride my bike more and when my daughter was going to school […], we walked to school […] the [pool] was pretty close and we'd go swimming there sometimes with her school and stuff.” Eric (a walkability decliner) expresses how the family facilities in his new neighbourhood allow him to be a role model for his children while staying active: “we play soccer at the local community centre with my children. And walk and play in the yard and local playgrounds […] I coach.” Relocating to a neighbourhood with amenities for winter activities encourages families to stay active all year long. Alexia (a walkability improver) lists the ways her new neighbourhood's community centre increases activity levels for herself and her family: “in the wintertime, there's a big arena […] we can skate and play hockey. […] we go tobogganing […] we get out a little bit more in our [new] neighbourhood because of that.”

Neighbourhood school transportation policies and walking routes influence the extent to which neighbouring families socialize. Victor (a walkability improver) describes his new neighbourhood as affording more opportunities to connect with other families and engage in physical activity since fewer children take the bus to and from school and the pathways are designed for safe travel with children: “In [our old neighbourhood], the majority of the kids were bussed in to go to school, so the playground would be empty after school and we didn't really get a chance to connect with a lot of families that lived [nearby]…. The pathway system [in our current neighbourhood] is connected right into the neighbourhood so [the kids] can access neighbourhoods without crossing a major street [which] was a barrier for them [in previous neighbourhood].” Enhancing safe family-friendly walking paths can engage entire families in transportation walking. However, living in heavily car-dependent neighbourhoods may pose barriers to physical activity with young children despite parents' intentions to be active with their children as Caroline (a walkability improver) notes “My daughter starts preschool and it's within a ten minutes walk […] so I'm thinking: how can I convince [my husband] to walk her there? But I think he's going to just drive her there ‘cause that'll be quicker.” Features like parks, playgrounds, tobogganing hills, skating rinks, pools, sports fields, low traffic residential streets, and paths to nearby schools are appreciated by parents for increasing opportunities to connect with members of their own family while participating in physical activity.

Some dog owners feel their physical activity increased through walking and connecting via their pet; Eva (a walkability improver) claims “Everyone should get a dog. It will change their lifestyle!” Owners see dog walking as a way of connecting with other dog owners in their neighbourhood as Victor (a walkability improver) expresses “we met a lot of our initial friends when we first arrived through obviously walking our dog and meeting dog walkers.” Participants mention built characteristics such as off-leash areas and parks as enablers of local dog walking.

*(2) Connecting to Nature through Sustainability*. Participants value physical activity in the form of active transportation for both the positive feelings they experience through connecting with nature noted above and for being able to give back to nature by defying car culture and reducing their carbon footprint. Victor (a walkability improver) expresses satisfaction with his family's active transportation supportive lifestyle as he describes having to work hard to secure this in the face of Calgary's pronounced car-friendly culture: “I think if we can promote this [active transportation] as a generation and pass it on to our kids' generation, then that is going to be a huge factor […] to reduce our footprint”. Those who participate in active transportation are often motivated by sustainability. Built characteristics that allow participants to connect with nature and future generations through their desire for sustainable transportation options (e.g., cycle and pedestrian pathways) support physical activity.

### 3.2.3. Adjusting or Adapting Physical Activity Behaviours in Response to a New Environment

Following residential relocation, participants adapted to opportunities within their new neighbourhood to stay active. For example, Eric (a walkability decliner) decreased transportation walking and cycling after relocation but also increased his overall physical activity. Yet Eric's new neighbourhood is closer to his work, and thus, his cycle commute is shorter which explains his reported decrease in transportation cycling. His new neighbourhood, albeit having fewer amenities than his previous more walkable neighbourhood, is more compatible with being physically active with his children as it is quieter, so he increased his overall physical activity, explaining “There's better playgrounds and a better park.” In other cases, the opposite is true, with participants who decreased their leisure physical activity following an increase in active transportation. Caroline (a walkability improver) explains “I probably do the same [amount of physical activity] because I do less walking of our dog, but [now] I walk to the train, whereas [in previous neighbourhood] we were too far from the train for me to walk.” Walkability improvement or decline does not always translate to respective increases or decreases in overall physical activity. Since some participants commute between neighbourhoods and do not attend destinations in their own neighbourhoods, neighbourhood walkability becomes less important for active transportation. In addition, support for transportation physical activity conferred by improved walkability does not always translate to support for leisure physical activity. Participants seek opportunities in their new neighbourhood environments for active transportation, leisure walking, and family activity or community sports and adjust their physical activity according to what is available and compatible with their lifestyle.

## 4. Discussion

Our hypothesis for transportation cycling was supported as on average, walkability decliners reported a slight decrease in transportation cycling, while walkability improvers on average reported little or no perceived change in their transportation cycling after relocation. Despite only approaching statistical significance, on average, walkability improvers reported a slight increase in transportation walking, while walkability decliners on average reported little or no perceived change in their transportation walking after relocation. We found no association between overall physical activity and walkability. These findings are consistent with a previous paper that included the same quantitative data with a different analysis (logistic regression) to compare walkability maintainers to those who relocated and increased or decreased walkability [40]. This previous study found that, compared to maintainers, walkability decliners decreased transportation walking, and walkability improvers increased transportation cycling. Our quantitative findings are supported by studies elsewhere showing increases in active transportation following increases in walkability [8–10]. Associations between Walk Score® and leisure walking are mixed with two studies finding no association [10, 53] and one study finding that lower Walk Score was associated with decreased leisure walking [54]. Similar to previous qualitative research, we found that greenery, aesthetics, safety and opportunities to connect motivated leisure walking and physical activity [19, 32, 34]. This finding may explain the absence of a relationship between overall physical activity and Walk Score® found in our quantitative analysis. Contrary to previous findings [27], we did not find that individuals changed their physical activity levels because of fear of crime. However, individuals did not express any significant changes in perceived crime rates following neighbourhood relocation, which may explain why fear of crime did not contribute to changes in physical activity in our study. A novel finding was that some participants described compensating their leisure time physical activity with active transportation following residential relocation to maintain overall physical activity levels. Notably, there is mixed quantitative evidence demonstrating compensation or trade-off between physical activity types [55–57].

Our qualitative findings suggest that neighbourhoods with more intersections and increased connectivity may encourage adults to walk for transportation; however, these same neighbourhoods will likely have few cul-de-sacs and potentially more vehicle traffic which could negatively impact perceptions of traffic safety and aesthetics and thus, might lead to less leisure walking. Renalds et al. also found that fewer street intersections (i.e., less connectivity) and lower traffic volumes as well as enjoyable scenery were positively associated with walking. Quantitative evidence regarding the association between connectivity and physical activity is sometimes mixed; however, these differences may be a physical activity outcome dependent (e.g., transportation vs leisure walking) [5, 58, 59]. Nevertheless, neighbourhood designs that combine grid-like street patterns but with cul-de-sacs or dead ends that stop vehicles but not pedestrians (i.e., “fused-grid” designs) could encourage more walking. Fused-grid neighbourhoods can support walking trips while discouraging car use [60] as well as reduce pedestrian exposure to air pollution and decrease risk of motor vehicle-pedestrian and bicyclist collisions [61].

In addition to built characteristics, factors such as stage of life, not quantified but presented in our qualitative data, influence physical activity decisions following residential relocation. Our qualitative findings suggest that future research on physical activity could be enriched by investigating physical activity of entire families as complementary units in interaction with their environment in their attempts to be physically active. Parents who resided in neighbourhoods that allowed them to stay active while working around their children claimed that having children enhanced their physical activity levels. Quantitative evidence shows that parents' physical activity levels and perceptions of the built environment can positively influence child physical activity levels [62–64]. At the same time, other parents expressed that having young children was a barrier to physical activity when they lived in neighbourhoods with scarce opportunities for family activities, and this finding is supported by quantitative evidence that found physical activity decreases in adults with children [65]. Our study suggests that neighbourhoods that sheltered children from high traffic areas and provided facilities for year-round outdoor activities (i.e., skating rinks, playgrounds, pools, and tobogganing hills) enhanced parents' physical activity by facilitating activity between children and caregivers. Parents' perceived neighbourhood safety from crime, availability of parks and playgrounds, decreased connectivity, increased aesthetics and walks, and cycle paths have been found to increase children's physical activity in previous research [63, 64], but increased children's physical activity does not necessarily translate to increased caregiver physical activity [64]. In addition to bonding with children, bonding with the family dog and other dog owners motivated participants to walk. This finding is congruent with previous research that found the dog owners are more likely to achieve recommended levels of physical activity [66, 67]. In the Calgary context, built characteristics such as aesthetics, walkability, nearby off-leash areas, and street pattern are found to be positively associated with frequency of dog walking [68, 69].

Our quasi-longitudinal mixed method study design included participants' retrospective self-reported change in physical activity, which likely was impacted by memory and recall bias. While the direction and relative magnitude of the change was reported, the absolute change in physical activity could not be ascertained. A longitudinal design where change in physical activity is captured, preferably using objective measures such as accelerometers, before and after participant's move may avoid such biases. The quantitative survey did not capture change in leisure walking because we focused on expecting change in built environment to have more influence on active transportation and less or no influence on recreational walking as has been found elsewhere [10, 53]. Our study does not account for time elapsed since relocation, and we only report changes in physical activity within the first 12 months of moving in the quantitative findings, which may miss changes that occur after 12 months of residence. Our qualitative findings account for changes within 3 years of moving. Notably, physical activity changes immediately after relocation may differ from changes that occur after a longer period of neighbourhood residence once the resident has adapted to their new surroundings [70]. We were unable to recruit an equal number of women and men, and thus, men's voices are underrepresented in our findings. Our small sample of movers used in the quantitative analysis also limits the generalizability of our findings. Nevertheless, our study's emphasis was on the qualitative findings, which used the quantitative findings in support. A strength of our study is the use of mixed methods and our qualitative analysis of narratives, which enabled in-depth insights into what changes in neighbourhood environment influence context-specific physical activity.

## 5. Conclusions

Our qualitative analysis added important context to our quantitative findings. These findings are innovative in that we found no studies bringing together qualitative and quantitative data in adults who changed physical activity habits following a change in neighbourhood and thus taps into an existing knowledge gap. Specifically, we found that more walkable neighbourhoods may explain increases in transportation walking and cycling. However, participants may compensate changes in transportation walking and cycling with increases or decreases in leisure physical activity to maintain similar overall physical activity levels—not found to be associated with walkability. Leisure physical activity included both leisure walking and cycling as well as activities that allow participants to form connections with family, community, and nature. These findings have implications for urban and transportation planners and policymakers. Specifically, urban and transportation policies and design that reduce local motorized traffic (e.g., fused-grid street patterns), support public transportation, and create direct and connected walking and cycling paths separated from motorized traffic to nearby destinations could support improvements in transportation walking and cycling. For adults who reside in less walkable neighbourhoods, built environment features that encourage leisure physical activity such as natural aesthetic elements, interesting places to explore, and facilities such as large parks, soccer fields, playgrounds, and skating rinks could also help increase physical activity. Neighbourhoods, where many young families reside, could leverage the caregiver relationship to promote physical activity among adults and children through the creation of family-friendly community activities, especially in those neighbourhoods with urban designs that are less supportive of walking.

## Figures and Tables

**Table 1:** Sociodemographic characteristics by the residential relocation group.

Sociodemographic characteristics		Residential relocation group		*p* value^*∗*^
		Moved to less walkable neighbourhood (*n* = 49) “decliners”	Moved to more walkable neighbourhood (*n *= 48) “improvers”	
		Estimate^a^	Estimate^a^	
Age, years (SD)		41.64 ± 16.67	42.83 ± 15.59	0.72
Have children <18 years, %		20.4	35.4	0.10
Sex, %	Women	73.5	49.3	0.95
Highest education achieved, %	High school	14.3	10.4	
	College	16.3	18.8	0.83
	University	69.4	70.8	
Marital status, %	Married/common law	67.3	75.0	0.41
Dog ownership in past year, %	Owner	36.7	54.2	0.09
Motor vehicle access, %	Never/do not drive	12.2	6.3	0.31
Injury in the past year, %	No injury	65.3	79.2	0.13

^a^Percent estimated for categorical variables and mean estimated (SD) for continuous variables. ^*∗*^Pearson's chi-square estimated differences for categorical variables and *T*-test estimated differences for continuous variables.

**Table 2:** Differences in mean activity change by the residential relocation group.

Activity	Residential relocation group		
	Moved to less walkable neighbourhood “decliners” (*n *= 49)	Moved to more walkable neighbourhood “improvers” (*n *= 48)	*p* value^a^
	Mean ± SD	Mean ± SD	
Walking for transportation	2.96 ± 1.12	3.29 ± 0.87	0.053
Cycling for transportation	2.69 ± 0.96	3.02 ± 0.84	0.039^b^
Overall physical activity	3.29 ± 1.06	3.29 ± 0.97	0.489

^a^ *T*-test estimated differences for activity change means. ^b^Estimate is significantly different between groups at *p* < 0.05.

**Table 3:** Qualitative sample characteristics (*n* = 14).

Pseudonym	Age	Gender	Change in transportation walking since relocation	Change in transportation cycling since relocation	Change in overall physical activity since relocation
*Walkability improvers*					
Becky	23	Female	Increase	Same	Increase
Eva	30	Female	Increase	Increase	Increase
Alexia	45	Female	Increase^*∗*^	Same	Same
Caroline	32	Female	Increase^*∗*^	Same	Same
Chrissy	32	Female	Increase	Increase	Increase
Melina	33	Female	increase^*∗*^	Increase^*∗*^	Increase^*∗*^
Marie	48	Female	Increase	Increase	Increase
Denise	41	Female	Same	Same^*∗*^	Increase^*∗*^
Victor	43	Male	Increase	Increase	Increase

*Walkability decliners*					
Rosa	30	Female	Increase	Same	Increase
Eric	33	Male	Decrease	Decrease	Increase
Laura	56	Female	Decrease	Decrease	Same
Emilia	66	Female	Increase	N/A^*∗*^	Same
Bernadette	—	Female	Decrease	Decrease	Decrease

^*∗*^the participant changed their initial answer from the quantitative survey during the qualitative interview.

## Data Availability

The qualitative and quantitative data used to support the findings have not been made available due to restrictions from the participant consent. Qualitative participant quotations used to support the findings of this study are included within the article.
